# Service Evaluation of a Boxercise Programme in an Inpatient Rehabilitation Setting

**DOI:** 10.1192/bjo.2024.474

**Published:** 2024-08-01

**Authors:** Alexander Graham, Leah Canning, Meenakshi Lachman

**Affiliations:** Leeds and York Partnership Foundation Trust, Leeds, United Kingdom

## Abstract

**Aims:**

We undertook a service evaluation obtaining feedback from service users in an inpatient rehabilitation setting about a weekly Boxercise class. The aim was to assess the experiences of service users, and the role it has in their recovery.

We hypothesised that the class would be well received by service users in aspects of enjoyment, impact on biopsychosocial wellbeing and recovery based on positive comments made by service users.

There is an increasing trend to utilise physical activity as an adjunct to improve mental health within healthcare settings; to increase motivation, educate on healthier lifestyles and to enhance well-being outcomes. This Boxercise programme has been developed by the Healthy Living Advisor within the rehabilitation inpatient facility at Leeds and York Partnership Trust. The programme has run for one year, and there has been a large uptake of service users who participate in the group. The Boxercise classes aim to encourage discipline, communication, spatial awareness, and cognitive skills in a modality that is interesting to service users.

**Methods:**

Service users who are regular participants in a Boxercise programme at an inpatient rehabilitation centre completed a questionnaire. A five-point Likert scale assessed participant views across seven domains. Participants were then asked to write three words that describe their feelings about the Boxercise programme, complete a drawing showing their thoughts after a Boxercise class and provide suggestions for improvement.

**Results:**

Eleven participants completed the questionnaire. Average scores for the domains were as follows: enjoyability 4.45/5 (89%), physical health 4.55/5 (91%), mental health 4.27/5 (85%), recovery 4.09/5 (82%), socialising 3.91/5 (82%), safety 4.64/5 (93%), continue after discharge 3.36/5 (67%).

The ‘three words' were put in a word cloud generator with highest weighted words: ‘Fun', ‘Good', ‘Energetic', ‘Confident'.

Common themes from the pictures shown were smiling faces and ‘strongman' images.

Six participants gave feedback that more equipment (pads and gloves) would help to improve their experience in the classes.

**Conclusion:**

The Boxercise programme received positive feedback from participants that aligns with the hypothesis; particularly in safety, enjoyability, benefit to physical health and benefit to mental health.

The participants had positive views on the class as an adjunct to the management of their physical and mental wellbeing. The feedback from all the participants is that they felt safe during the classes.

This service evaluation indicates that the participants value the Boxercise classes as an enjoyable activity and as an adjunct to their treatment.

